# Hepatitis C-related cryoglobulinemic neuropathy: potential role of oxcarbazepine for pain control

**DOI:** 10.1186/s12876-018-0751-9

**Published:** 2018-01-25

**Authors:** Rita Moretti, Paola Caruso, Matteo Dal Ben, Silvia Gazzin, Claudio Tiribelli

**Affiliations:** 1 Neurology Clinic, Department of Medical, Surgical, and Health Sciences, University of Trieste, 34100 Trieste, Italy; 2 Italian Liver Foundation, Centro Studi Fegato, AREA Science Park, Bldg. Q, Ss 14, km 163.5, 34149 Trieste, Italy

**Keywords:** HCV, Oxcarbazepine, Neuropathy, Pain

## Abstract

**Background:**

Peripheral neuropathy is one most common, limiting and invalidating neurological symptom in subjects with hepatitis C virus and mixed cryoglobulinemia. Notably, the medical therapy proposed to eradicate HCV, can frequently exacerbate the painful neuropathy. Therefore, neuropathy therapies are insufficient and inadequate, and comprise immunosuppressive drugs, such as steroid or cyclosporine, intravenous immunoglobulin or plasma exchange. These have shown variable success in case reports, with a presumably temporary effect, but with major side effects.

**Methods:**

We assessed the effects of oxcarbazepine treatment in 67 cases of cryoglobulinemia related neuropathy, who did not respond to either steroid or Gabapentin, or Pregabalin. Oxcarbazepine was chosen based on the promising preliminary results.

**Results:**

Patients treated with Oxcarbazepine showed a rapid, discrete and persistent relief of polyneuropathic signs, without consistent side effects, and with a limited interaction with concomitant drugs.

**Conclusions:**

These data favor the use of oxcarbazepine as a useful tool in the management of neuropathic pain associated with Hepatitis–C cryoglobulin neuropathy.

## Background

Cryoglobulins are cold-precipitating immunoglobulins [1], which form aggregates and immune complexes, outstripping the reticuloendothelial-clearing activity, depositing in the human tissues, activating the complement, and finally leading to tissue damage, promoting systemic inflammation (usually occurring at the level of skin or kidneys). Otherwise healthy individuals could present cryoglobulins, making difficult to estimate their real prevalence in medical sets, finally considered rare. Nevertheless, the variable clinical manifestations reported in literature, might leads to underestimation of cryoglobulinemia diffusion.

Cryoglobulins can be divided into three types, following the Brouet classification [2], essential, or secondary to autoimmune pathologies and chronic medical conditions (primarily chronic infections) and related to lymphoproliferative diseases [2]. The second and third types, also defined as mixed cryoglobulins [2, 3], composed by monoclonal Ig rheumatoid factor and polyclonal IgG and IgM rheumatoid factor, are seen very frequently in HCV infections [3–7].

Despite varying from country to country, the prevalence of peripheral neuropathies in HCV-associated cryoglobulinemia substantially varies in the Mediterranean [1, 3, 8]. Of note, HCV-related proteins are present in damaged skin, blood vessels, and kidneys and seem to play a direct role in the pathogenesis of the damages seen in the peripheral nerve [1, 9, 10].

Pathological findings suggest a direct damage of the small vessels around nerve, the so-called *vasa nervorum* [3, 11, 12], due to a vasculitis or to necrotizing arteritis. Both these conditions cause an ischemic damage of nerve [3, 10, 13, 14]. HCV is directly responsive for inducing the inflammation cascade of events in the vessels, since it has been demonstrated HCV-RNA in epineurial cells [15–17].

Peripheral neuropathy is the most common reported symptom in patients with HCV-associated mixed cryoglobulinemia [18–20], where it may be the first clinical manifestation, despite its prevalence is still unknown. There is only a report [14] assessing the prevalence of peripheral neuropathy in HCV; neuropathy was found in 33% of patients without other cryoglobulinemia-related symptoms and clinically, most frequently, patients present a symmetrical sensory or motor-sensory polyneuropathy, or less frequently as a mono-neuropathy, or as a multiple mononeuropathy [3, 15].

At biopsy, axonal degeneration is shown, possibly triggered by the deposit of cryoglobulins at the level of *vasa nervosum* microcirculation and vasculitis-induced ischemia, as well, despite seldom reported, by an immunological mediated demyelination. Electrophysiological studies and teased nerve fiber studies allowed neuropathies to be classified as predominantly sensory axonopathies [21, 22], even if there are some descriptions of demyelinating peripheral neuropathies [3, 21–24].

There is no conventional treatment for HCV cryoglobulin neuropathy. It is accepted nowadays that prolonged antiviral therapy led to a reduction of HCV-RNA levels, associated with a reduction of cryoglobulinemia [25]. The reduction of cryoglobulins under the detectable levels has been obtained by treating patients with interferon-alpha (IFN alpha) plus ribavirin, obtaining an amelioration of painful polyneuropathy. On the other hand, the side effects of IFN alpha therapy are known, with the exacerbation of the symptoms of mixed cryoglobulinemia, with an exacerbation of the neuropathy, whit severe myalgia, arthralgia [26, 27]. Moreover, there are different cases of described side effects of IFN alpha, such as demyelinating sensory neuropathy, neuropsychiatric symptoms, a possible bone marrow dyscrasia, a transient or definite worsening of hepatitis [22, 28, 29]. For cryoglobulin-related neuropathy, other options have been proposed, including alternative immunosuppressive agents, such as steroid or cyclosporine [30], and plasma exchange [31]. They showed variable success and the effect is presumably temporary. There are some works which stressed the importance of steroids and cyclophosphamide [12]. Considering that peripheral cryoglobulin-related neuropathy in HCV patients give, as the most frequent symptom, the neuropathic pain, the principle target should be its earliest relief and without (or with the most limited) side effects.

After the promising results of a previous work [11], we present a series of many patient, with HCV cryoglobulinemia related polineuropathy, who did not respond to steroid and Gabapentin treatment, but have been successfully managed with Oxcarbazepine.

## Methods

### Patients

Sixty-seven HCV-positive patients (details in Tables 1 and 2) followed by the Liver Center of the University of Trieste from 1st January 2000-to 1st January 2015, have been studied in the Neurology Unit due to the detection of peripheral neuropathic signs. All the patients were treated with IFN-αlpha and ribavirin therapy, three times a week; a progressive decrease of their viral load was observed in all of them. Neurological signs of peripheral neuropathic signs appeared for 11 patients 9.7 ± 2.1 months after the cessation of antiviral therapy even with a sustained viral response, and increase in the cryoglobulins serum level. Forty-four patients interrupted antiviral therapy (mean period of treatment of 7.6 9.7 ± 2.1) due to neurological symptoms such as anxiety and depression (18 patients), suicidal thoughts (6 patients), major sleeping disturbances, concentration difficulties and daily living executive complications (8 patients), apathy, chronic fatigue, loss of weight (12 patients). Drop out are in line with some data presented in Literature, i.e. by Manns et al. [32, 33] All the 44 patients showed cryoglobulin neuropathic polyneuropathy 3.4 ± 1.2 months after stopping antiviral therapy. Twelve patients were strained to interrupt the IFN and ribavirin therapy (after mean time of therapy duration of 6.2 ± 3.9 months) due to the appearance of painful peripheral neuropathy, associated with an elevation of cryoglobulinemia, and therefore excluding the IFN-related neuropathy.

**Table 1:** Epidemiological and biological features in 67 patients with HCV and cryoglobulinemia

Patients (total 67)	Features
Age (years)	50.4 ± 2.7
Sex ratio (M/F)	31/36
Apparent duration of disease (years)	4.1 ± 1.3
Albumin level (g/L)	39.7 ± 8.5
ALT (IU/L)	131.4 ± 16.7
Prothrombin time (%)	84.1 ± 12.2
Rheumatoid factors (n of pts. and %)	41/67 (61%)
Mean Cryoglobulinemia(g/L)	0.27 ± 0.8
Cryoglobulinemia type	
CGS TYPE II (n of pts. and %)	42/67 (64%)
CGS TYPE III (n of pts. and %)	25/67 (36%)
ANTI HCV Antibodies (n of pts. and %)	67/67 (100%)
ANTI HBV Antibodies (n of pts. and %)	35/67 (52%)
Anti HBC	30 (86%)
HBsAg	5 (14.2)
Anti HBS	30 (86%)
HBeAG	0
AntiHBe	0
HCV RNA sequences in sera (n of pts. and %)	49/67 (73%)

**Table 2:** HCV status of the 67 patients

Patients (total 67) (number and %)	Features
Anti HCV antibodies	67/67 (100%)
Chronic Hepatitis	67/67 (100%)
Cirrhosis	6/67 (8.9%)
Presumed disease duration (years)	6.1 ± 2.7
Fibrosis score	1.9 ± 0.7
HAI score [47]	6.5 ± 1.2
HCV genotype (number and %)	
1	43 (64.1%)
2	1 (1.5%)
3	21 (31.3%)
4	2 (3%)

The new antiviral drugs (sofusbuvir, simeprevir, daclatasvir, etc.) were not used, since at the time of the recruitment they were not available. All of the 67 patients reported progressive painful paresthesias, shock pain episodes, boot-glove dysesthesia sensations. All the patients showed signs of cryoglobulinemia (mean 0.27 +/− 0.8 g/L; range 0.05–1.6 g/L). Cryocrit levels were measured as the percentage of packed cryoglobulins after cold centrifugation of the serum, and cryoglobulin composition was determined by immunodiffusion on Ouchterlony plates against specific antisera. Fifty-eight had polyneuropathy and 9 had mononeuropathy multiplex. All subjects had an electroneurographic confirmation of peripheral neuropathy, 57 presented the axonal profile and 10 showed the axonal-demyelinating signs of damage (Table 3). Thirty-six patients referred a relatively acute onset of pain and numbness in feet and legs together with a rash on the dorsal surface of low limbs, in particular, starting from the feet, extending midway up to the legs. Seventeen patients presented with the acute onset of pain, described as “electric shocks” which is followed by numbness in legs. These symptoms started approximately two years prior presentation of overt liver disease, and gradually worsened. Fourteen patients (all women) presented a mainly sensory neuropathy, asymmetrical, with dysesthesia symptoms and were committed due to a presumptive restless leg syndrome, which was not confirmed by the neurologist. All the patients denied any other symptom such as fever, chills, arthralgias or skin alterations, apart from those described above, or other general hepatic-referable symptoms; 24 patients admitted alcohol consumption before the beginning of IFN-alpha and ribavirin therapy.

**Table 3:** Electrophysiological and clinical features of the examined patients

Symptoms	Patients
Neuropathy type	
Polyneuropathy	58
Mononeuropathy	9
Electrophisiological features	
Demyelination	10
Axonal	57

Reduced complement C4 activity has been found in 39 patients; positive search for antinuclear antibodies has been found in 13 patients, anti-mitochondrial in 19 patients and C-reactive protein has been found in 32 patients.

### Pharmacological treatment

**Fig. 1:**
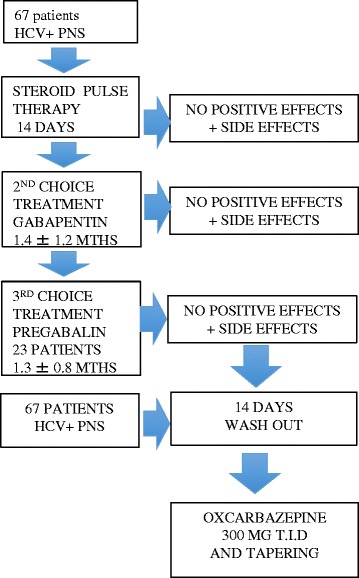
Patients flowchart

As shown in Fig. 1, after the electroneurographic confirmation of the clinical diagnosis of cryoglobulinemia related polineuropathy, patient started a cycle of steroids for 14 days, followed by Gabapentin if steroids failed to improve the simptoms. If Gabapentin treatement was not successful or accompained by side effects, it was substituted with Pregabalin, witch was soon interrupted for severe side effects. After one month of wash-out, all the 67 patients, who still complained for the neurological sympotms began Oxcarbazepine. A neuriological assesemsnt was performed every month for 12 months after the start of Oxcarbazepine treatement.

### Statistical analyses

Statistical analyses were performed using SAS® software (version 16.0 SAS® Software Inc., Cary, NC, USA). Within-group changes from baseline were tested using the Wilcoxon Signed Ranks test. Between-group comparisons of changes from baseline were tested using the Marginal Homogeneity Test. Spearman correlation test has been employed for each significant variable. This was done for each efficacy variable. Results are presented as mean changes from baseline with standard deviations, and *p*-values are provided where appropriate.

## Results

As in Fig. 1, after the clinical diagnosis all patients were treated with steroids (up to 100 mg/daily of prednisone); 10 patients partially responded, 40 experienced steroidal side effects, which causes interruption, and 17 did not show any benefit.

One month after the steroid washout, gabapentin. It was prescribed (1887 ± 205 mg/day) followed by a moderate improvement in the painful sensation (average visual analog scale from 8 to 9 to 6–7). After one month of follow-up, due to daytime sleepiness, nausea, dizziness and gait alteration the drug was stopped. The average duration of the treatment was 1.4 ± 1.2 months.

In 23 strongly motivated patients, pregabalin (50 mg, t.i.d) was prescribed; 14 patients showed cholestatic and liver damage, 2 presented overt jaundice and 7 patients a prolongation of the prothrombin time. All side effects subsided after the medication was stopped. No amelioration of pain symptoms was observed.

At the end of this period of treatment (average time of 5.6 months), all the 67 patients reported side effects and persistence of pain. After two weeks of washout of any other pain-relief drug(s), oxcarbazepine was given at a dose of 300 mg/day for a week, increased to 600 mg/day the following week, and then further increased to 900 mg/day. The dosage was increased to 1200 mg/day in 11 patients for 10.2 ± 2.3 days, due to persistence of pain, to be then readjusted to 900 mg/day. Each patients was follow-up monthly for 12 months, and each check-up, the Short form McGill Pain Assessment Questionnaire (SF-MPQ) [34] was administered. The average SF-MPQ was 32.1 ± 5.7 at baseline (average scores 24–36), and decreased to 21.3 ± 2.3 (average scores 17–25, *p* < 0.001) after one month. The average SF-MPQ at 6-month was 12.5 ± 3.7 (average scores 9–14; p < 0.001 over baseline). At the 6th month of follow-up, the dose of Oxcarbazepine was reduced to 600 mg/day; 33 patients were pain free and maintained the dose for two additional months while 34 increased to 900 mg/day. All the 67 patients, who have been prescribed Oxcarbazepine, completed the 12 months follow-up: 46 patient arte asymptomatic and do not take any anti-epileptic drug. All patients referred sleepiness and drowsiness at the beginning of the therapy that rapidly disappeared; no other side effects were observed.

## Discussion

Hepatitis C is a serious health concern, affecting millions of people worldwide. The majority of the subject positive for HCV antibody are asymptomatic. More recently, central (CNS) and peripheral nervous system (PNS) have been studied as focal collateral damage regions, tightly involved in HCV.

The treatment of HCV-cryoglobulins -associated PNS is actually based IFN plus ribavirin therapy, eventually associated with steroids [4, 35–37]. Studies have showed that patients with HCV-associated cryoglobulinemia and extra-hepatic manifestations treated with IFN-alpha alone were reported to be responders (resolution of symptoms, disappearance of cryoprecipitates and HCV RNA), with only one tapering off of IFN-alpha after 3 years of treatment with sustained resolution of HCV and cryoglobulinemia [34]. However, long-term studies were not performed and the results of long term follow-up are not available [38–40]. The few data available seems to indicate favorable outcomes in cryoglobulinemia HCV subjects treated with corticosteroids [10, 39, 40]. Since chronic steroid administration also increases the level of HCV RNA, this treatment should be limited. Therefore, intravenous immunoglobulin or plasmapheresis should be considered as a therapeutic option [24]. In addition, in patients with severe cryoglobulinemia-associated vasculitis as those with rapidly progressive renal failure or neurological involvement, the antiviral therapy should be delayed for 2–4 months while they are treated with aggressive scheme with plasmapheresis, high doses of corticosteroids and either cyclophosphamide or rituximab [41]. Rituximab therapy has been used predominantly in HCV-related mixed cryoglobulinemia refractory to or unsuitable for corticosteroids and antiviral (IFN-α) therapy [41]. The role of interferon to exacerbate cryoglobulinemia related neuropathy is still under evaluation, although recent data obtained in 24 patients showed that interferon-free regimen with new drugs as sofosbuvir resulted in an almost complete clinical response of the vasculitis but still undefined effect on pain relief [42].

Painful neuropathy is a dramatic and persistent condition, which affects many patients, and is an invalidating condition. The several options available for pain control are confusing and daunting. The best solution seems to be an escalating regime matching the intensity and nature of the sensory components of the pain state. Moreover, in chronic conditions, such as HCV- related hepatitis, where many different clinical details should be considered, pain relief therapies should not interfere with the underline disease and the concomitant antiviral treatment. Therefore, pain-treatment should be efficacious, should not interfere with their general and hepatic condition, should not create side effects as, in particular, disequilibrium, gait imbalance, drowsiness and reduced vigilance.

Oxcarbazepine is a safe drug, without significant side effects, and the rarely described hepato-toxicity seems to be related to a hypersensitivity or to a immune –mediated response to some metabolites of the drug, rapidly and totally reversible [43]. Oxcarbazepine should be careful monitored by specialists, with reported side effects, which can be distinguished in: *more common, but rapidly and spontaneously solving side effects (1:100):* Blurred vision, disequilibirum, mental depression, emotional incontinence, cough and sore throat sensation. *Less common (less than 1:1000):* Agitation, awkwardness, mental confusion, persistent disequilibrium associated with orthostatic hypotension, fast or irregular heartbeat, thirst, muscle cramps, headache, skin rash, weakness. Usually they spontaneously disappeared, and they do not need a suspension of therapy. *Rare (less than 1: 3500)*: Anxiety, burning feeling in the chest or stomach, hives or itching, irritability, restlessness, muscle pain or weakness, purple spots on the skin and rectal bleeding; these needs a careful medical examination, and the consequent decision to stop it.

Oxcarbazapine possess intrinsic pain-relief properties [44], either considering acute or chronic pain [44, 45] and neuropathic pain [46]. The efficacy of previous studies, its safety, the good tolerance and the limited side effects leads to this study, which confirmed the previously reported data [10]. Results are stable, we have not reported serious adverse side effects and pain relief is stable. Our study demonstrated that treatment of HCV-cryoglobulins related neuropathy with oxcarbazapine is effective in the large majority of patients at a dosage which is rather well tolerated and is not associated with any relevant side effect. The particular condition of these patients (particularly vulnerable to hepatotoxicity), the conspicuous potential interactions with concomitant drugs taken by the patients (antiviral, etc), the rapid beneficial effect (demonstrated by the decrease of the average SF-MPQ), and the absence of consistent side effects (demonstrated by a good pattern of labs values) favour to the useof oxcarbazepine in the management of neuropathic pain.

## Conclusions

In this work, patients not responsive to steroids, gabapentin at high dosage and to pregabalin, benefit from oxcarbazepine. The limited side effects, associated to the favourable and rapid response, the reduced interaction with concomitant drugs support the use of oxcarbazepine as an effective and safe treatment of the of HCV-cryoglobulins related neuropathy.

## Abbreviations

CNS: Central nervous system
GGT: Gamma-glutamyltranspeptidase
HAV: Hepatitis A virus
HBV: Hepatitis B virus
HCV: Hepatitis C virus
IFN: Interferon
IFN-alpha: Interferon-alpha
MHD: 10-hydroxcarbazepine
PNS: Peripheral nervous system
SF-MPQ: Short form McGill Pain Assessment Questionnaire
VAS: Visual analogue scale
